# Differential Regulation of Neutrophil Elastase Release vs. NET Generation After Cardiopulmonary Bypass

**DOI:** 10.3390/ijms27156634

**Published:** 2026-07-25

**Authors:** Erin Tresselt Murray, Jessica S. Hook, Parth Patel, Lihua Xie, Jessica Moreland

**Affiliations:** 1 Division of Critical Care Medicine, University of Texas Southwestern Medical Center, 5323 Harry Hines Blvd, Dallas, TX 75390, USA; 2 Department of Pediatrics, Children’s Medical Center Dallas, University of Texas Southwestern Medical Center, Dallas, TX 75390, USA; 3 Department of Microbiology, Children’s Medical Center Dallas, University of Texas Southwestern Medical Center, Dallas, TX 75390, USA

**Keywords:** neutrophil, inflammation, neutrophil extracellular trap, cardiopulmonary bypass, organ injury

## Abstract

Cardiopulmonary bypass (CPB) during heart surgery provokes an inflammatory response that can cause post-operative organ injury, yet how CPB shapes key neutrophil effector programs is unclear. We investigated whether CPB differentially regulates neutrophil degranulation versus neutrophil extracellular trap (NET) formation and whether pre-operative neutrophil phenotypes associate with organ dysfunction. In a prospective cohort (*n* = 39) of children (1 day–4 years) undergoing CPB, blood samples were obtained before CPB and 24 h post-operatively. Purified neutrophils were analyzed for elastase activity and NET formation, alongside phenotypic markers of activation and neutrophil–platelet aggregation. Findings were correlated with acute kidney injury, respiratory failure, and cardiovascular dysfunction. CPB induced marked neutrophilia (4.5-fold rise; *p* < 0.0001). Elastase release was robustly enhanced after CPB and showed limited augmentation with secondary stimulation but did not predict organ dysfunction. In contrast, NET formation was significantly reduced after CPB. Importantly, diminished pre-operative responsiveness to low-dose NET priming was observed in patients who developed organ injury. Pediatric CPB uncouples neutrophil effector programs, priming degranulation while suppressing NETosis. Pre-operative NET “reserve” may define a clinically relevant innate immune endotype at risk for post-operative organ dysfunction and supports development of biomarker-driven treatment strategies in patients undergoing CPB.

## 1. Introduction

Children with congenital heart disease often require surgical repair, which frequently includes the use of cardiopulmonary bypass (CPB) during the procedure. Although there have been many advances in CPB over the past 75 years, CPB still presents unique challenges in children due to their immature immune systems and heightened inflammatory responses resulting in organ dysfunction in a subset of cases [1,2]. Around 50% of patients experience some degree of organ injury after CPB with a subset of patients experiencing severe inflammatory complications [3]. Those with single- or multiorgan injury often require prolonged hospitalization and demonstrate increased morbidity and mortality [4]. Previous work has shown that kidney injury is associated with increased mortality and length of ICU stay in patients post-CPB [5]. We currently lack a predictive model to indicate which patients will experience life-threatening inflammatory issues post-CPB. Post-operative cardiac surgical care is primarily algorithmic without a precision approach to protect from organ injury. Advancement in the quality of outcomes will require a better understanding of the molecular and cellular mechanisms driving inflammatory complications in order to develop targeted individualized therapies for this vulnerable population.

The adaptive immune response has been extensively studied in order to describe inflammatory complications after CPB [6]. However, there are far fewer studies defining mechanisms of innate immune involvement including the role of neutrophil activation responses. The focus of most inflammatory complications after CPB has been on the role of monocytes and macrophages rather than neutrophils. Neutrophils play a pivotal role in host defense mechanisms and are the most numerous leukocytes in circulation and often the first leukocyte to respond to sites of infection or injury. They are armed with an arsenal of highly toxic compounds that they can deploy within minutes of stimulation which play a crucial role in amplifying the immune response. In a previous pilot study from our laboratory [7], we found that patients who had neutrophils displaying enhanced NADPH oxidase activity after CPB were 8.8 times more likely to develop acute kidney injury (AKI) compared to patients who had less Nox2 activity after CPB, independent of the length of CPB.

In other disease processes, neutrophil products have been demonstrated to be relevant in organ injury including elastase release, NET formation, inflammatory cell surface markers, and cytokine production [1,8,9]. Our lab previously demonstrated that in patients with COVID-19, NET formation early in the course of illness correlated with severity of lung disease [10]. We chose to focus on elastase and NET formation in CPB patients because multiple clinical trials targeting elastase and NET components are underway using the molecular mechanisms underlying these neutrophil responses [11,12,13,14].

The goal of the current study was to evaluate neutrophil function to provide further insight into the pathophysiology of CPB-related inflammation to allow consideration of therapeutic targets in the future. The long-term goal is to provide individualized care to patients at risk of organ injury. Surprisingly, we found that all patients had an increase in elastase release and a decrease in NET formation, but these were not predictive of organ injury.

## 2. Results and Discussion

### 2.1. Patient Enrollment

To assess neutrophil inflammatory phenotype, a total of thirty-nine patients were enrolled in this study out of 150 patients that were screened. Screening occurred by reviewing the cardiac surgical list each Monday for eligible patients. Patients’ parents were approached unless surgery was scheduled on days where samples could not be processed in the lab. Patients were excluded if they had a known neutrophilic disorder or neutropenia. The demographic data for our enrolled patients are displayed below in Table 1.

Half of our patient population identified as Hispanic. We had a broad variety of cardiac operative procedures for a tertiary CVICU ranging from STAT categories 1–5, the majority being STAT category 2 (44%), and 41% were STAT categories 3 or higher. Initial blood samples of 33% of patients were taken before anesthesia, and 66% of initial blood draws were taken 15 min after anesthesia induction.

We assessed basal neutrophil phenotype and neutrophil phenotype pre- to post-CPB by several different assays that assess PMN activation to see if neutrophil phenotype was predictive of end-organ injury including acute kidney injury (AKI), acute respiratory failure (RF), and cardiovascular (CV) dysfunction.

### 2.2. Enhanced Neutrophil Number Following Cardiopulmonary Bypass

**Figure 1:**
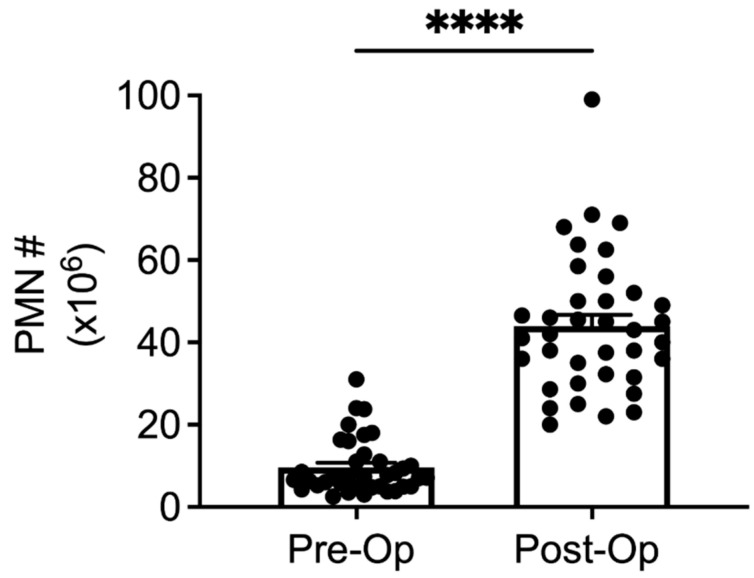
Neutrophil production increases significantly pre-operatively to post-operatively. Neutrophil number measured pre-operatively to post-operatively. In all patients, the average absolute number of neutrophils increased by 4.5-fold following cardiopulmonary bypass (**** = *p* value < 0.0001).

First, we looked at the overall quantity of neutrophils pre- to post-bypass with the hypothesis that neutrophil function plays a role in inflammation after cardiopulmonary bypass. We found that in all patients, the number of neutrophils significantly increases from pre-operative to post-operative, as shown in Figure 1. The average absolute neutrophil count increased by 4.5-fold following cardiopulmonary bypass compared with pre-operative levels. Although total WBC also increased from the pre-operative period to the post-operative period, the increase in PMN count was approximately five-fold greater than the increase in the total WBC count, reflecting a shift in the leucocyte differential. Given the enhanced mobilization of total neutrophils, we next sought to determine if these cells displayed enhanced activation markers.

### 2.3. Surface Expression of L-Selectin and ICAM-1 Not Altered by Cardiopulmonary Bypass

**Figure 2:**
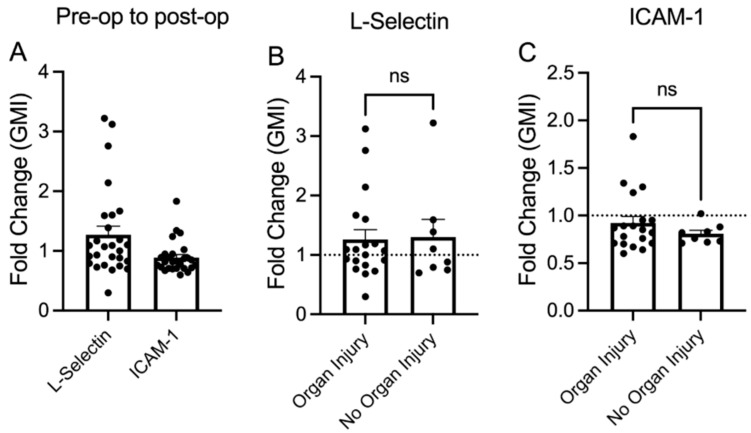
L-selectin and ICAM-1 expression do not significantly change with CPB and are not predictive of organ injury. L-selectin and ICAM-1 expression do not significantly change with CPB and are not predictive of organ injury. (**A**) displays a broad distribution of L-selectin levels, but the trend for higher L-selectin post-bypass was not consistent with what would be seen with shedding. ICAM-1 ex-pression was low, but unchanged with CPB. (**B**,**C**) highlights that there is no significant change in the surface expression of L-selectin or ICAM-1 in patients who have organ injury compared to those who do not by looking at the fold change in GMI (post-op/pre-op) where a value of 1 means there was no change, greater than 1 means the expression increased, and less than 1 means it decreased. ns, not significant.

Several cell membrane surface markers can be measured as surrogates for neutrophil activation. We elected to study surface expression of L-selectin and Intracellular adhesion molecule (ICAM-1). L-selectin is often shed during inflammatory processes and ICAM-1 is increased on the cell surface, and both proteins are relevant to the neutrophil recruitment cascade in inflammatory states [15]. Both L-selectin and ICAM-1 expressed by neutrophils have been studied in human inflammatory diseases and are used as a quick-change marker of inflammatory states. Although there was a fairly broad distribution of L-selectin levels, the pre- to post-bypass fold change was not significantly different, and the trend was for higher L-selectin surface expression in the post-bypass cells, rather than lower as would be seen with shedding (Figure 2A). ICAM-1 expression was quite low, but unchanged post-bypass. We analyzed the pre-to-post change in neutrophils from patients that would later display organ injury in comparison to those without organ injury. Neither L-selectin nor ICAM-1 levels were predictive of future organ injury (Figure 2B,C). These data suggest that the enhanced neutrophil number seen following CPB is not accompanied by a change in activation state. Given that cell surface marker expression is not the only measure of PMN activation, we elected to study neutrophil–platelet aggregation post-bypass and in the setting of organ injury.

### 2.4. Platelet Aggregation Trends Lower Post-Operatively

**Figure 3:**
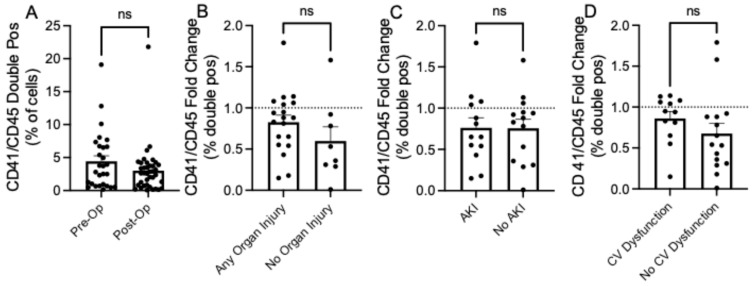
Flow cytometry was utilized to measure neutrophil platelet aggregation trends pre- to post-operatively from cardiopulmonary bypass. Neutrophil–platelet aggregate number was used to correlate between organ dysfunction and the number of aggregates. (**A**) highlights that there is a trend towards decreased neutrophil–platelet aggregates pre-operatively to post-operatively, but it is not statistically significant. There is also a trend towards increased platelet aggregation in patients who go on to develop any organ injury as seen in (**B**), but it is not statistically significant. (**C**,**D**) highlights a trend of increased neutrophil–platelet aggregates in patients with AKI or CV dysfunction, but again it is not significant. Neutrophil–platelet aggregates were measured by CD41 and CD 45 positivity. ns, not significant.

Platelets also serve as inflammatory mediators and are relevant to the pathogenesis of organ injury in patients undergoing cardiopulmonary bypass [16,17]. We used flow cytometry to determine if there was a correlation between subsequent development of organ injury (AKI, RF, and/or CV dysfunction) and a higher number of neutrophil–platelet aggregates using double positive cells, known to represent these aggregates. We found that there was no significant change in the number of aggregates from pre- to post-evaluated cells. Moreover, there was no significant finding of double positive cells and association with organ injury (Figure 3). We hypothesize that this finding may reflect neutrophil–platelet aggregates that are sequestered in the organs, causing damage in the microcirculation, but not present or measurable in the peripheral blood.

### 2.5. Neutrophil Elastase Secretion Is Enhanced After CPB but Does Not Predict Organ Injury

**Figure 4:**
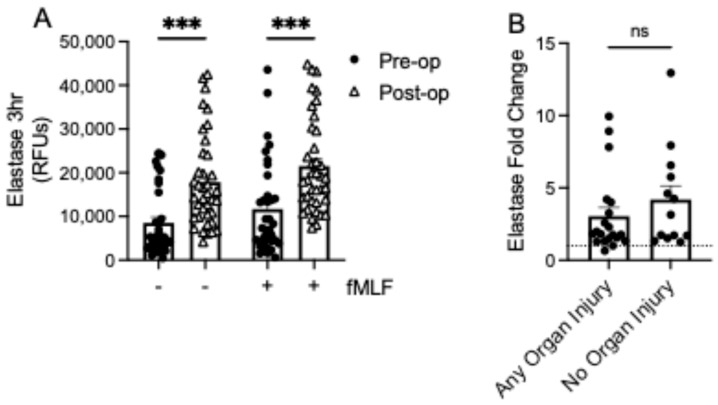
Neutrophil elastase activity in response to fMLF increases pre-operatively to post-operatively but does not trend with organ injury. (**A**): Utilizing an elastase functional assay, exposure to CPB alone elicited robust neutrophil elastase release when comparing elastase in pre- vs. post-CPB cells; however, there was no significant difference in the post-op CPB patient neutrophils when stimulated with fMLF (*** = *p*-value < 0.001). (**B**) shows there were no significant differences in elastase release in patients who would go on to have organ injury (AKI, CV, or RF). ns, not significant.

We next focused on the exocytosis of primary granules, using elastase secretion as a surrogate for mobilization of this granule type. Neutrophil secretion of elastase and other primary granule contents can enhance the inflammatory environment and cause tissue damage. Elastase release has been implicated in ARDS and other respiratory failures, and clinical trials have been initiated demonstrating the relevance of these measurements [18,19,20]. We sought to assess the impact of CPB on patients’ neutrophil elastase release under baseline and stimulated conditions. Using an elastase functional assay, we noted that exposure to CPB alone elicited robust neutrophil elastase release when comparing elastase in pre- vs. post-CPB cells (Figure 4A). Using N-formyl-methionyl-leucyl-phenylalanine (fMLF), a known neutrophil priming stimulus to stimulate cells, we saw no significant difference in the post-op CPB patient neutrophils, compared with pre-op, suggesting the cells were already primed by the bypass exposure. Subgroup analysis of these data demonstrates that there were no significant differences in elastase release in patients who would go on to have organ injury (AKI, CV, or RF), when compared to those who would not have organ injury (Figure 4B).

### 2.6. NET Formation Diminished After CPB, but Not Predictive of Organ Injury

**Figure 5:**
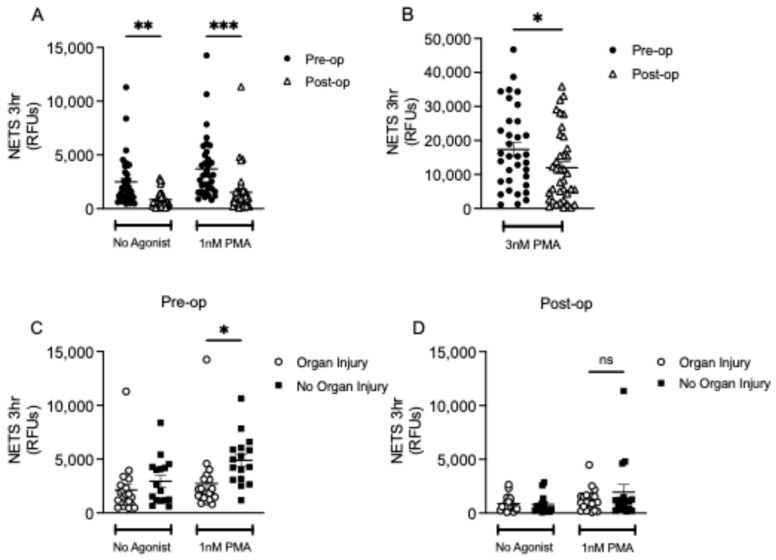
Net formation measurement in pre- and post-operative neutrophils. Using Sytox Green, (**A**,**B**), shows decreased NET formation in all patients when stimulated with 1 nM PMA or 3 nM PMA when comparing pre-operative to post-operative conditions (** = *p*-value < 0.01; *** = *p*-value < 0.001). In (**C**), patients who would later display organ injury (post-op) had significantly diminished pre-op NET formation in response to lower concentrations of PMA, whereas all cells had impairment in the post-op condition (**D**). (* = *p*-value < 0.05). ns, not significant.

Given the phenotypic results in elastase measurement, we next focused on NET generation. NET formation has been implicated in numerous and varied disease pathologies ranging from Rheumatoid Arthritis to COVID-19 [9,21,22]. It is known that several unique signaling pathways result in NET formation. Our lab recently optimized an assay to measure elastase activity and NET formation simultaneously using very few cells. Based on our interest in better understanding these two distinct neutrophil responses and their respective roles in organ injury, we measured NET formation and NET formation potential (ex vivo) response to exogenous stimulation with stimuli known to induce NET formation) in pre- and post-CPB neutrophil samples. Surprisingly, we found that exposure to CPB resulted in significantly decreased NET formation in patients after CPB when compared to before CPB (Figure 5A,B). This was true both in unstimulated conditions and in response to low or high concentrations of PMA, a well characterized stimulus of NET formation. Intriguingly, cells from patients who would later have organ injury displayed significantly diminished pre-op responsiveness to priming/lower concentrations of PMA (Figure 5C), whereas all cells had impairment in the post-op condition (Figure 5D).

## 3. Discussion

Cardiopulmonary bypass remains essential for repair and palliation of congenital heart disease, and although there have been great strides in outcomes, it still exposes children to a potent “sterile” inflammatory insult that can result in multiorgan dysfunction. Early mechanism-related biomarkers to predict which patients will develop inflammatory complications are not available. In this context, neutrophils are uniquely positioned at the interface of innate immune sensing and vascular injury: they are rapidly mobilized after extracorporeal circulation and can execute divergent effector programs (adhesion, degranulation, NET formation) with distinct implications for host defense versus collateral tissue damage [23,24,25]. Accordingly, defining which neutrophil programs are engaged by CPB—and whether pre-operative neutrophil “endotypes” presage organ injury—has practical importance for risk stratification and for developing targeted immunomodulatory strategies that go beyond nonspecific anti-inflammatory approaches.

In this prospective cohort of pediatric patients undergoing cardiac surgery with CPB, we performed paired pre- and post-CPB neutrophil phenotyping using complementary assays to capture PMN-based inflammation and relate it to subsequent organ injury endpoints (AKI, respiratory failure, and/or cardiovascular dysfunction). Consistent with CPB-driven systemic inflammation, neutrophil abundance increased markedly following bypass, outpacing the overall leukocyte rise and indicating a substantial differential shift. Notably, this quantitative surge was not accompanied by conventional activation-marker changes: neither L-selectin nor ICAM-1 showed significant pre-to-post fold change, and neither predicted later organ injury. Similarly, circulating neutrophil–platelet aggregates did not increase post-operatively and were not associated with organ injury. Intriguingly, two functional observations stand out as the most novel findings of this study. First, CPB exposure was associated with robust neutrophil elastase release (a surrogate of primary granule exocytosis), and subsequent (fMLF) stimulation did not further augment elastase release—supporting a “primed-by-CPB” state—yet elastase output did not predict which patients would develop subsequent organ injury. Second, and unexpectedly, NET formation and NET inducibility decreased after CPB across conditions. Specifically, diminished pre-operative responsiveness to low-dose PMA priming characterized patients who would later develop organ injury. Taken together, these results suggest that in pediatric CPB, neutrophil effector programs may be selectively reprogrammed rather than uniformly “activating” all classical inflammatory axes.

However, the conclusions that can be drawn are limited by a population with wide variability in age range and included diverse surgical operations and level of complexity (STAT categories 1–5, with substantial representation of higher complexity cases). Moreover, the cohort was not of adequate size to allow multivariable adjustment, or account for residual confounding by bypass duration, hypothermia strategy, transfusion burden, steroid exposure, anesthesia exposure, and peri-operative infection risk.

NETs merit particular attention because they represent a mechanistically rich and clinically actionable intersection of innate immunity and vascular pathology. Beyond antimicrobial containment, NETs can propagate endothelial injury and amplify complement and coagulation [26,27,28,29,30]. In critical illness, circulating NET components (e.g., cell-free DNA, histones, MPO–DNA complexes) have been linked to microvascular dysfunction and organ injury in multiple inflammatory syndromes (sepsis, trauma), and in extracorporeal settings the combination of contact activation, shear stress, ischemia–reperfusion, and transfusion exposures plausibly promotes NET-relevant signaling [22,31,32]. Our finding that ex vivo NET formation capacity falls after CPB, despite evidence of priming for degranulation, raises several possibilities. CPB may drive (i) rapid intravascular NET deployment with subsequent “consumption” or sequestration of NET-competent neutrophils into tissues, (ii) induction of counter-regulatory pathways that blunt NETosis, or (iii) compartmentalization wherein NET formation occurs primarily within organ microcirculations rather than in peripheral blood—aligning with our inability to detect increased circulating neutrophil–platelet aggregates. Importantly, the observation that diminished pre-operative NET responsiveness to low-dose PMA associates with later organ injury is potentially a marker of NET “reserve capacity”.

The present data underscore functional heterogeneity: although all patients experienced a striking post-operative neutrophil expansion, canonical surface-marker readouts (L-selectin/ICAM-1) were comparatively uninformative for predicting downstream injury, whereas functional readouts (elastase release, NET inducibility) revealed a more nuanced reprogramming. From a bedside perspective, this is attractive because pediatric CVICU decision-making depends on early discrimination of children at highest risk for AKI, prolonged ventilation, or cardiovascular instability—often before creatinine rises, radiographic findings evolve, or vasoactive needs fully declare themselves.

Finally, several additional limitations for interpretation of this study should be discussed. First, sample size and the possibility of selection bias impacted the power to detect associations between immune phenotypes and relatively infrequent outcomes. Second, our analyses relied on peripheral blood phenotyping; inability to detect increased circulating neutrophil–platelet aggregates do not preclude clinically relevant sequestration within lung or kidney microvasculature, and similarly, reduced ex vivo NET formation after CPB could reflect post-bypass functional exhaustion of circulating cells rather than reduced NET activity in tissues. Lastly, 67% of patient samples were collected 15 min following induction of general anesthesia, which may have influenced signaling and neutrophil function. Therefore, anesthesia-related immune modulation cannot be excluded as a contributor to the observed findings [33].

## 4. Materials and Methods

### 4.1. Study Patients

This was a prospective cohort study conducted in a tertiary Cardiovascular Intensive Care Unit (CVICU) performed from September 2019 to September 2022. During this period, enrollment was halted for 6 months during the outbreak of the COVID-19 Omicron variant. Institutional Review Board approval was obtained by UT Southwestern Medical Center and site approval by Children’s Health. Inclusion criteria were patients with congenital heart disease undergoing repair with CPB aged one day old to four years old. Patients were excluded if they had a known neutrophilic disorder or neutropenia. The parents of the patients signed written consent the day before or morning of surgery.

### 4.2. Data Collection

Peri-operative and post-operative data were collected including age, sex, race, diagnosis, STAT (The Society of Thoracic Surgeons-European Association for Cardio-Thoracic Surgery) category, and respiratory support. Data were collected for each patient for the first four post-operative days or until the patient was discharged from the CVICU, whichever came last.

Data collection focused on three endpoints of inflammation-induced organ injury: acute kidney injury, acute respiratory failure, and cardiovascular dysfunction. AKI was calculated based on the KDIGO (Kidney Disease Improving Global Outcomes) classification. KDIGO stages AKI severity from Stage 1 to 3 based on the level and rate of serum creatinine increase and total urine output, with Stage 1 being mild and Stage 3 severe. [34].This was calculated pre-operatively and at 24 and 48 h post-operatively. Fluid overload was also estimated on each patient for four days post-operatively, which was calculated by in/out balance (liters) post-operatively divided by the pre-operative weight (kg) and multiplied by 100.

To assess respiratory failure, total ventilator days, non-invasive mechanical ventilation (including bilevel positive airway pressure (BiPAP)) and high-flow nasal cannula (HFNC) days, and non-ventilatory days were counted. Respiratory failure was defined as the requirement for invasive or non-invasive mechanical ventilation >5 days. Cardiovascular dysfunction was assessed by the validated Vasoactive-Inotropic Score (VIS), which is an objective tool that is used to measure the amount of cardiovascular support in children after cardiac surgery [35]. VIS was calculated day one to two post-operation. Cardiovascular dysfunction was assigned to patients with a VIS greater than 10 on post-operative day one or persistently greater than 6 for 2 consecutive days. Each of these organ dysfunction endpoints were correlated with neutrophil functional and phenotypic analyses.

### 4.3. Blood Collection

Samples were collected in heparin anti-coagulated tubes using universal precautions from each patient at two points for this prospective study. For patients enrolled without invasive lines prior to surgery, the baseline sample was collected in the operating room within 15 min of induction of general anesthesia prior to the onset of surgery and use of CPB. For patients already with an arterial line or central line, the baseline sample was taken right before the patient was taken to the operating room. The second sample was then collected 24 h after surgery while the patient was in the CVICU. All patients except one have pre-operative and post-operative samples. Blood samples were immediately processed for neutrophil isolation and evaluation of neutrophil function and phenotype.

### 4.4. Neutrophil Purification Process

Patient neutrophil isolation was completed according to standard techniques from heparin anti-coagulated venous or arterial blood samples in accordance with a protocol approved by the IRB and included stepwise isolation using dextran sedimentation followed by separation by density gradient using Hypaque-Ficoll, and completed with erythrocyte lysis using hypotonic solutions as previously described [36,37]. The cell concentration was determined by manually counting using a hemacytometer. Cell purity was assessed by examining a stained slide microscopically and was found to be greater than 98%. Trypan blue exclusion was used to assess cell viability immediately prior to experimental setup and was found to be greater than 99%.

### 4.5. Flow Cytometry Analysis of Cell Surface Protein Expression

Neutrophils were analyzed using a FACScalibur flow cytometer from BD Biosciences (Franking Lakes, NJ, USA). Flow cytometry was used to measure cell-surface expression of proteins associated with neutrophil–platelet aggregation (CD41/45) and proteins associated with cell activation, including L-selectin and ICAM-1. Freshly isolated neutrophils were fixed with 4% paraformaldehyde on ice for 30 min, followed by a 20 min incubation in ice-cold antibody diluting buffer (PBS with 2% milk and 4% normal goat serum) and staining with fluorophore-conjugated primary antibodies (eBioscience 17-0411-82 for CD41, eBioscience 11-0451-82 for CD45, BD 562330 for L-selectin, and BD 560971 for ICAM-1) according to the manufacturer’s specifications. Following incubation on ice for 60 min, cells were washed and resuspended in PBS. Flow cytometry was then performed. Size (forward scatter; FCS) and granularity (side scatter; SSC) were used to gate on the neutrophil population. Given the purity of the cell isolation, gating was primarily used to exclude debris. For CD41/45, data are presented as the % double positive as compared with cells stained with isotype control or the fold change in % double positive. L-selectin and ICAM-1 are reported as the fold change in geometric mean intensity from pre-op to post-op. Flowjo analysis software, version 10.0.08, from TreeStar (Ashland, OR, USA) was used for flow analysis.

### 4.6. Measurement of Elastase Activity

Exocytosis of azurophilic granules was measured using a plate-based assay of extracellular elastase activity. Isolated PMNs were diluted in buffer containing 7-amino-4-methylcoumarin (AMC), a fluorogenic, proteolytic substrate of elastase. The PMNs were placed in a 96 half-well plate and some were incubated with TNF-α at time 0. At 30 min, the PMNs were injected with fMLF as a weak stimulus of granule mobilization. Peak relative fluorescence units at 90 min, a previously established technique [38], was measured by a CLARIOstar plate reader from BMG Labtech (Cary, NC, USA).

### 4.7. Neutrophil Extracellular Trap Formation

Sytox Green: Freshly isolated PMNs were incubated in a 96 half-well plate with 5 µM Sytox Green with specified stimuli in HBSS containing 0.1% glucose and 1% human serum albumin. Fluorescence was measured using the CLARIOstar from BMG Labtech (Cary, NC, USA) at an excitation of 491 +/− 15 nM and emission of 535 +/− 20 nm with readings every 5 min for six hours. In priming experiments, 1 µM fMLF or 1nM PMA (Phorbol 12-myristate 13-acetate) was injected following a 30 min incubation time with different stimuli. Kinetic and endpoint readings are expressed as relative fluorescence units (RFUs).

### 4.8. Statistical Analysis

Data were analyzed using GraphPad Prism version 11.0.2 (100) We assessed priming of elastase activity with and without TNF-α compared to the amount of elastase release pre- to post-CPB at 90 min. NET formation was measured by looking at the pre- to post-CPB total amount of NET release at 5 h. Analysis of variance (ANOVA) and Student’s *t*-test were used where appropriate.

Conclusions: In conclusion, despite several described limitations, the findings in this study are important, suggesting that pediatric CPB does not simply “turn on” neutrophils uniformly; instead, it may differentially tune distinct effector programs. Importantly, baseline NET hyporesponsiveness may identify a subgroup predisposed to organ injury. Future directions include: (1) validating the pre-operative NET-reserve signal in a larger, multicenter cohort with standardized organ injury definitions and multivariable modeling; (2) adding longitudinal sampling (pre-CPB, immediate post-CPB, 6–24 h, and 48–72 h) to map the trajectory from hyperinflammation to immune dysfunction and link it to ventilator days, renal replacement therapy, and nosocomial infection; (3) integrating functional assays with circulating biomarkers (plasma elastase activity, MPO–DNA complexes, cell-free DNA, DNase activity), coagulation parameters, and endothelial injury markers to resolve whether reduced NET capacity represents/sequestration versus active suppression; and (4) mechanistic studies examining how CPB exposures (heparin/protamine, hypothermia, circuit coatings, transfusion products) shape neutrophil signaling pathways that partition degranulation from NETosis.

## Figures and Tables

**Table 1:** Demographic data for patients enrolled in this study.

Variables	Patients
Age (months) (% patients)	
<12 months	85%
>12 months	15%
Sex (% Male)	56%
STAT Category (% patients)	
1	15%
2	44%
3	8%
4	18%
5	15%
Patients with Acute Kidney Injury (AKI %)	38%
Patients with Respiratory Failure (RF %)	20.5%
Patients with Cardiovascular dysfunction (CV %)	31%
Patients with Multiorgan Dysfunction (MODS %) (2+systems)	23%

## Data Availability

The data that support the findings of this study are available from the corresponding author upon reasonable request.
